# Comparison of liver volumetry on contrast‐enhanced CT images: one semiautomatic and two automatic approaches

**DOI:** 10.1120/jacmp.v17i6.6485

**Published:** 2016-11-08

**Authors:** Wei Cai, Baochun He, Yingfang Fan, Chihua Fang, Fucang Jia

**Affiliations:** ^1^ Department of Hepatobiliary Surgery (I) Zhujiang Hospital Southern Medical University Guangzhou Guangdong China; ^2^ Research Lab for Medical Imaging and Digital Surgery Shenzhen Institutes of Advanced Technology Chinese Academy of Sciences Shenzhen Guangdong China

**Keywords:** liver volumetry, CT image, image segmentation, evaluation

## Abstract

This study was to evaluate the accuracy, consistency, and efficiency of three liver volumetry methods— one interactive method, an in‐house‐developed 3D medical Image Analysis (3DMIA) system, one automatic active shape model (ASM)‐based segmentation, and one automatic probabilistic atlas (PA)‐guided segmentation method on clinical contrast‐enhanced CT images. Forty‐two datasets, including 27 normal liver and 15 space‐occupying liver lesion patients, were retrospectively included in this study. The three methods — one semiautomatic 3DMIA, one automatic ASM‐based, and one automatic PA‐based liver volumetry — achieved an accuracy with VD (volume difference) of −1.69%,−2.75%, and 3.06% in the normal group, respectively, and with VD of −3.20%,−3.35%, and 4.14% in the space‐occupying lesion group, respectively. However, the three methods achieved an efficiency of 27.63 mins, 1.26 mins, 1.18 mins on average, respectively, compared with the manual volumetry, which took 43.98 mins. The high intraclass correlation coefficient between the three methods and the manual method indicated an excellent agreement on liver volumetry. Significant differences in segmentation time were observed between the three methods (3DMIA, ASM, and PA) and the manual volumetry (p<0.001), as well as between the automatic volumetries (ASM and PA) and the semiautomatic volumetry (3DMIA) (p<0.001). The semiautomatic interactive 3DMIA, automatic ASM‐based, and automatic PA‐based liver volumetry agreed well with manual gold standard in both the normal liver group and the space‐occupying lesion group. The ASM‐ and PA‐based automatic segmentation have better efficiency in clinical use.

PACS number(s): 87.55.‐x

## I. INTRODUCTION

The incidence of hepatocellular carcinoma (HCC) is rising with more than 800,000 deaths per annum.^(1)^ Currently, surgical operation, locoregional therapy, extracorporeal energy therapy, and systemic therapy are the main treatment methods. However, for surgically resectable patients, liver transplantation and partial hepatectomy have been considered as the main curative treatment.^(2)^ The accurate preoperative measurement of liver volume is essential for successful surgical operation.^3^, ^4^, ^5^ Evaluation of total and segmental liver volumes has become crucial for adult living donor liver transplantation (LDLT) because the graft size is a major factor in predicting the safe outcome for both donor and recipient.^(6)^ An accurate and noninvasive liver volumetry is also vital to determine the remaining functional liver volume in HCC patients receiving hepatectomy.

Computed tomography (CT), as the main imaging modality for liver surgical planning, plays an important role in quantitative radiology and precision surgery.^7^, ^8^, ^9^ Manual volumetry on CT images is the current “gold‐standard” for liver volume calculation. Although manual volumetry can deliver a relatively accurate result, the lengthy and tedious operation, subjective determination, and intraobserver and interobserver disagreement discourage its usage in routine clinical work. Semiautomatic interactive liver volumetry methods are relatively less time‐consuming than the manual volumetry, and they have been widely used in clinical practice.^(10)^ Doctors can correct a certain degree of error and obtain a relatively accurate result by manual postprocessing procedures. With the rapid growth of machine learning and image analysis techniques, highly accurate automatic volumetry methods may substitute for the manual or semiautomatic in clinical liver volume calculation. Some studies on volumetry methods have been conducted in recent years. Nakayama et al.^(11)^ compared automated with manual liver volumetry and found that there was no significant difference (p=0.407); however, they found that automatic liver volumetry was more difficult to perform in severely damaged livers than healthy livers. Linguraru et al.^(9)^ compared normalized probabilistic atlas segmentation with manual volumetry in 77 normal cases and 71 hepatomegaly cases; the automatic liver volumetry validated was accurate, but there were no cases with space‐occupying lesions or distinct appearance differences to compare with the normal datasets. Suzuki et al.^(12)^ validated an automatic liver extraction approach on 18 normal prospective living liver donors; the volume difference (VD) was 0.070±0.047 and the correlation coefficient was 0.94. An automatic liver volume measurement software was evaluated with an accuracy of VD=0.040, but the automatic and manual volumetry method were statistically different (r=0.989, p<0.001).^(13)^ The above studies validated their automatic volumetry methods with gold standard, but they all have common shortcomings. Almost all the CT images they chose came from normal subjects or enlarged livers (hepatomegaly). Furthermore, automatic liver volumetry has not intensively been validated in disease models such as space‐occupying lesions, including liver tumors or hepatolithiasis, and there have been no comparisons between normal groups and space‐occupying lesion groups. Since liver lesions are quite common in routine clinical practice, the idea of doing this study was conceived.

The aim of this study is to evaluate the effectiveness of the three tools on our clinical datasets (including normal and space‐occupied diseased cases) through comparing their results with those calculated from the manual volumetry. The three tools include a semiautomatic interactive three‐dimensional medical image analysis (3DMIA) system, (a.k.a., MI‐3DVS — medical image three‐dimensional visualization system),^14^, ^15^ an active shape model (ASM)‐based segmentation,^(16)^ and a probabilistic atlas (PA) registration‐based segmentation method.^(17)^ The ASM and PA were validated in the Segmentation of the Liver 2007 challenge (SLIVER07: http://www.sliver07.org). The ASM method got a score of 75.5 and the PA method got a score of 72.4. A score of 75 was supposed to be as good as human observers.

## II. MATERIALS AND METHODS

### A. Case selection

Between April 2013 and January 2014, 889 consecutive patients were examined with abdominal 64‐slice multidetector CT (64‐MDCT) scanning in the Department of Radiology. A random‐number table was used to choose 5% cases from the database to constitute a study pool. Two of

them were excluded from the study pool due to the incomplete abdominal images acquired. Of the 42 cases finally selected, 27 cases are a normal liver group (group A, 21 men and 6 women) and 15 cases are a disease group (group B, 9 men and 6 women). In group A, the mean age of the men was 50.0±15.0 years (range, 5–81 years) and that of the women was 57.8±10.9 years (range, 41–70 years). In group B, the mean age of the men was 48.3±15.3 years (range, 24–72 years) and that of the women was 60.2±13.6 years (range, 38–77 years).

The disease group includes seven cases of HCC and eight cases of intrahepatic calculi. The characteristics of patients with liver lesions are shown in Table 1. Portal venous phase images were used in this study because they can maximize the intensity difference between liver parenchyma and nonliver tissue. The institutional review board (IRB) approved this study. Since this was a retrospective study, informed consents were not required.

**Table 1:** The characteristics of patients

*Patients with HCC (7 cases)*		*Patients with Intrahepatic Calculi (8 cases)*	
*Variables*	*Numbers*	*Variables*	*Numbers*
*Tumor Sites*		*Stone location*	
Single	6	Left lobe	4
Multiple	1	Right lobe	2
*Tumor location*		Bilateral	2
Left lobe	4		
Right lobe	2	*Atrophy*	
Bilateral	1	Left lobe	4
*Tumor size*		Right lobe	1
Xx<3 cm	1	Bilateral	1
3–10 cm	5	None	2
≥10 cm	1		

### B. Image acquisition

Data were collected using a Philips Brilliance 64‐MDCT scanner (Philips Medical Systems, Fitchburg, WI). Enhanced CT scanning was performed as follows: dynamic abdominal triphasic tomography and thin‐slice scanning were performed on the patients after nonionic iopamiro, an intravenous contrast agent, was administered. Each patient received 80 to 100 mL of iopamiro. The contrast was injected at a rate of 5 mL/s, followed by vascular flushing with 40 to 50 mL normal saline at the same rate. Arterial‐phase scanning was achieved by contrast agent tracing. Specifically, the scanning was automatically triggered 8 s after the vascular CT value in the diaphragmatic section of the abdominal aorta reached 100 HU. Portal venous phase scanning was initiated by the same criterion, but with a 60‐s delay. The scanning covered the area from the diaphragm to the lower poles of the kidneys. The scanning parameters included a voltage of 120 KV, a current of 200 mA, a detector combination of 0.625×64 mm, a pitch of 0.894, a bed speed of 47.5 mm/s, and a rotation time of 0.5 s. The reconstructed images were in 512×512×156∼512 in size and had an x‐axis and y‐axis spacing of 0.50–0.85 mm and slice thickness of 0.5–1.0 mm.

### C. Manual volumetry

To establish the ground truth reference standard, an experienced surgeon of hepatic surgery with a record of over 300 manual volumetry operations performed the manual volumetry. The liver volumes contoured were further double‐checked by another board‐certified radiologist to ensure the quality. Because the results were performed with perfect precision, the radiologist did not do any correction. The time required to complete the manual tracing for each case was recorded.

### D. Semiautomatic liver volumetry

The semiautomatic interactive segmentation exploits a geodesic active contour model, according to image gray‐scale and spatial information, to delineate the liver boundary.^18^, ^19^ The liver segmentation was also performed by the surgeon who conducted the manual liver volumetry. The technique of this method is detailed in Appendix A.

### E. Automatic liver volumetry

Two methods — ASM‐based deformable model and PA nonlinear image registration‐based — automatic liver segmentation were used. ASM and PA have gained widespread popularity because prior knowledge is available. The training process and parameters have been established by the developers. So the liver segmentation is fully automatic when performed by the surgeon. A surgeon just needs to input the image and wait until the output appears. The details of the two methods are described in Appendix B and C, respectively.

### F. Statistical analysis

The results obtained with automatic liver volumetry were compared with those obtained with the semiautomatic interactive volumetry and the manual volumetry using three metrics: live volume, relative volume difference (VD), and segmentation time. VD is defined as the volume difference of the automatic or interactive segmented set S and the gold standard reference set R,VD=(|S|−|R|)/|R|. The Dice coefficient DICE=2(|S|∩|R|)/|R|+|S| was also used for evaluating the segmentation accuracy.

SPSS 20.0 software (IBM Corp., Armonk, NY) was used to perform the statistical analysis. A p‐value less than 0.05 was considered statistically significant. When the test of homogeneity of variances was not significant (p>0.05), we performed an analysis of variance (ANOVA). When the test of ANOVA was not significant (p>0.05), we chose the least significant difference (LSD) or Bonferroni test to do the pairwise comparison.

When the test of homogeneity of variances was significant (p<0.05), we performed the Kruskal Wallis test. Finally, when the test of Kruskal Wallis was significant (p < 0.05), we chose the multiple Student's *t*‐test with Bonferroni correction to further compare the two sets of data.

Linear association was evaluated with the Pearson correlation coefficient (r). Intraclass correlation coefficient (ICC)^(20)^ was chosen to evaluate the agreement between automatic volumetry or semiautomatic interactive volumetry and manual volumetry. The interobserver agreement between the manual‐tracing surgeon and the double‐checked radiologist was also calculated. The evaluation of the level of agreement between two volumetric methods was performed by the method described by Bland and Altman.^(21)^

## III. RESULTS

As shown in upper half of Table 2, in normal group (group A), the mean volume obtained by manual volumetry, by semiautomatic 3DMIA, by automatic ASM‐based and by PA‐based volumetry was $1203.3\pm 285.6\;{\text{cm}}^{3}$ (range, $488-2,065\;{\text{cm}}^{3}$), $1182.4\pm 279.3\;{\text{cm}}^{3}$ (range, $481-1,997\;{\text{cm}}^{3}$), $1170.2\pm 279.9\;{\text{cm}}^{3}$ (range, $464-1,968\;{\text{cm}}^{3}$), and $1233\pm 274.5\;{\text{cm}}^{3}$ (range, $548-2,071\;{\text{cm}}^{3}$), respectively. Compared with the gold standard manual volumetry, the VD error was −1.69% with 3DMIA, −2.75% with ASM, and 3.06% with PA. The Dice coefficients of the three methods are 0.934, 0.923, and 0.936, respectively. The comparisons of liver volumes in the disease group (group B) are summarized in lower part of Table 2. Those results indicated that the three methods achieve accurate liver segmentations. The multiple comparison of liver volumes obtained by different methods were very similar without statistically significant differences: 3DMIA vs. manual in group A (p=0.783) and in group B (p=0.731), ASM vs. manual in group A (p=0.665) and in group B (p=0.696), PA vs. manual in group A (p=0.696) and in group B (p=0.710), ASM vs. 3DMIA in group A (p=0.875) and in group B (p=0.963), PA vs. 3DMIA in group A (p=0.505) and in group B (p=0.475), and PA vs. ASM in group A (p=0.410) and in group B (p=0.447). The multiple comparison of VD error among 3DMIA, ASM, and PA showed that the 3DMIA, ASM, and PA did not have statistically significant difference: ASM vs. 3DMIA in group A (p=0.233) and in group B (p=0.066), PA vs. 3DMIA in group A (p=0.715) and in group B (p=0.262), and PA vs. ASM in group A (p=0.406) and in group B (p=0.326).

**Table 2:** The results obtained by manual volume, 3DMIA, ASM, and PA in groups A and B

		*Mean (cm* ^*3*^ *)*	*Standard Deviation (cm* ^*3*^ *)*	*VD Error (%)*	*DICE Coefficient*	*Average Time (min)*
	Manual	1203.3	285.6	‐	‐	41.78±10.09
	3DMIA	1182.4	279.3	−1.69±4.31	0.934±0.035	27.63±4.50
*Group A*	ASM	1170.2	279.9	−2.75±4.01	0.923±0.013	1.28±0.51
	PA	1233.2	274.5	3.06±5.49	0.936±0.013	1.19±0.20
	Manual	1272.3	313.2	‐	‐	−47.93±6.32
	3DMIA	1233.1	317.4	−3.20±2.72	0.925±0.034	27.53±4.00
*Group B*	ASM	1228.2	300.0	−3.35±1.96	0.922±0.012	1.23±0.60
	PA	1314.4	301.6	4.14±8.34	0.924±0.02	1.16±0.09

In Table 2, for group A and group B, manual volume takes 41.78±10.09 and 47.93±6.32 min, respectively; 3DMIA takes 27.63±4.50 and 27.53±4.00 min, respectively; ASM takes 1.28±0.51 and 1.23±0.60 min, respectively; and PA takes 1.19±0.20 min and 1.16±0.09 min, respectively. There were significant differences between 3DMIA and manual volumetry, between ASM and manual volumetry, between PA and manual volumetry, between ASM and 3DMIA, between PA and 3DMIA, both in group A and in group B (all p<0.001). However, there were no statistically significant differences between ASM and PA in group A (p=0.950) or in group B (p=0.960).

**Figure 1:**
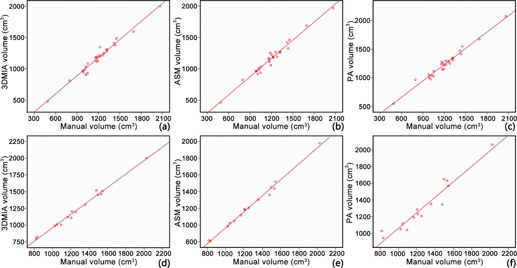
The relationships among 3DMIA, ASM, PA, and manual volumetry (reference standard): (a) volume measured with 3DMIA vs. gold standard in group A; (b) volume measured with ASM vs. gold standard in group A; (c) volume measured with PA vs. gold standard in group A; (d) volume measured with 3DMIA vs. gold standard in group B; (e) volume measured with ASM vs. gold standard in group B; (f) volume measured with PA vs. gold standard in group B.

**Figure 2:**
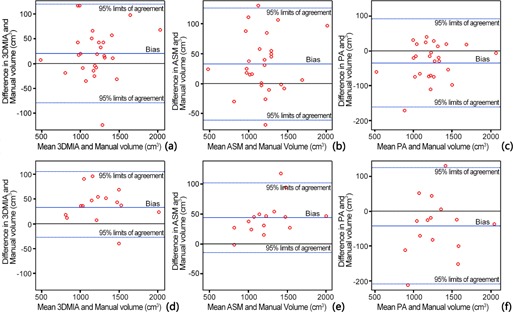
Bland‐Altman plots for agreement among different methods: (a) 3DMIA and manual volumetry in group A; (b) ASM and manual volumetry in group A; (c) PA and manual volumetry in group A; (d) 3DMIA and manual volumetry in group B; (e) ASM and manual volumetry in group B; (f) PA and manual volumetry in group B.

Figure 1 and Table 3 give the result of intraclass correlation coefficients and Pearson's correlation coefficients between different methods. The results indicate that there exists linear association between the different methods. Table 4 and Fig. 2 give the results of the Bland‐Altman analysis. In Fig. 2, most points fall in the 95% limit‐of‐agreement confidence region. All those results indicate that, in both groups, 3DMIA, ASM, and PA liver volumetry achieved excellent agreement with manual volumetry.

**Figure 3:**
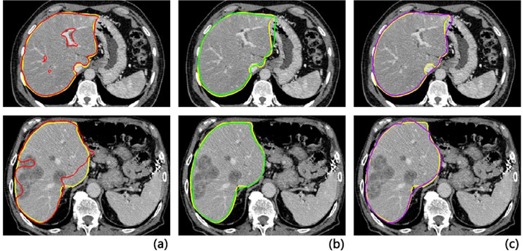
Comparison of segmentations between the three methods and the corresponding gold standard (yellow curve). Liver contour determined by (a) semiautomatic software (red), (b) ASM method (green), (c) PA method (purple). Normal case (top row) and diseased case (bottom row). Transversal views.

In Fig. 3, the liver boundaries extracted by semiautomatic interactive and automatic algorithms agreed with the manually segmented gold standard. In 3DMIA method, the branches of the portal vein were excluded from the liver area. In ASM method, the hard constraint of a shape model was in the region of the stomach, which was not completely excluded. PA also had a slight problem of oversegmentation in the area of the stomach and inferior vena cava. In the bottom row of Fig. 3, the automatic algorithms still showed accurate performances in diseased livers, whereas the semiautomatic method yielded a moderately inaccurate segmentation in need of interactive revision.

The interobserver agreement between the manual‐tracing surgeon and the double‐checked radiologist was 0.961.

**Table 3:** The results of intraclass correlation coefficient (ICC) and Pearson's correlation coefficient in groups A and B

		*Intraclass Correlation Coefficient*	*Pearson's Correlation Coefficient*
	3DMIA vs. manual	0.982	0.984
*Group A*	ASM vs. manual	0.980	0.986
	PA vs. manual	0.978	0.984
	3DMIA vs. manual	0.987	0.994
*Group B*	ASM vs. manual	0.985	0.996
	PA vs. manual	0.955	0.962

**Table 4:** The results of Bland‐Altman analysis in groups A and B

		*Bias (cm* ^*3*^ *)*	*95% Limits of Agreement (cm* ^*3*^ *)*
	3DMIA vs. manual	27.43	(−62.36,117.21)
*Group A*	ASM vs. manual	37.05	(−45.45,119.55)
	PA vs. manual	−34.24	(−160.90,92.42)
	3DMIA vs. manual	21.07	(−78.42,120.56)
*Group B*	ASM vs. manual	33.11	(−60.40,126.62)
	PA vs. manual	−29.89	(−130.18,70.40)

## IV. DISCUSSION

We achieved an excellent agreement in volume measurement between ASM vs. the gold standard, PA vs. the gold standard and 3DMIA vs. the gold standard in both group A and group B. Those results from each of the different methods were not significantly different. The volume error rate of different methods from different groups was superior to the previously reported volume error rate: 5.2%±4.2% from interactive and 7.0%±4.7% from automated methods^(12)^ and to the SLIVER07 standard for the VD threshold, which was set to 4.7% — that being believed accurate enough for comparison with an independent human observer.^(22)^

The ICC and Pearson correlation coefficients showed that, compared to manual volumetry, the three methods had an excellent correlativity when dealing with the same case. The results of Bland‐Altman analysis suggested that the three methods were all useful for routine clinical practice as almost all the dots are in the range of 95% confidence interval in six figures. The intraobserver variability comparison showed that the ICCs between 3DMIA, ASM, PA, and manual volumetry in the normal group (approximately equal to 0.980), and in the disease group (approximately equal to 0.986) were higher than the interobserver agreement. The ICC between PA and manual volumetry was slightly lower (0.955) than the interobserver agreement. All ICCs were comparable with the state‐of‐the‐art liver volumetry methods.^(12)^

It is well known that image appearance has an impact on the liver segmentation. In some cases, the left lobe of liver can be very large, making it difficult to distinguish the border between the liver and the spleen. In manual segmentation, boundaries can be found through observing many slices from CT images very carefully. In most situations, 3DMIA would reconstruct the liver and spleen at the same time, and the operator can extract the liver through the image postprocessing by watching the original CT images. The contrast is generally used to improve the intensity of tumors and hepatic vessels for later disease detection. Without the contrast, the liver shows more consistent intensity values and this will make the liver segmentation easier, as seen from the increased time of manual segmentation from the normal group to abnormal. But if contrasts are used, the 3DMIA is sensitive to tumors and hepatic vessels; thus, 3DMIA requires postprocessing to fulfill accuracy.

In contrast, ASM and PA have advantages in this respect due to the prior constraints. In combination with prior knowledge, the two automatic methods were robust to complex conditions, such as different CT scan phases, healthy and diseased livers and adjacent tissues. The ASM method captured the shape of liver from CT images robustly, but was prone to undersegmentation constrained by the prior shape model. Another limitation of ASM was that inadequate training samples have an impact on forecasting ability. For the PA method, the registration was more accurate if the target and the reference images were similar, but PA was prone to oversegmentation because the registration will move to the heart or the stomach, which may have a similar intensity range as the liver.

There are two limitations in this study. Only one person was involved in manual volumetry. But the manual‐tracing surgeon underwent lots of training and the correlation between him and the experienced radiologist was 0.961, which may imply that the interobserver disagreement may be ignored. Another limitation is the small number of liver lesions evaluated. That may limit variation among cases, implying insufficient data to ensure the accuracy and reliability of our results.

## V. CONCLUSIONS

In summary, accurate computerized liver extraction is still very challenging to perform due to the liver being in close relation with other organs of similar intensity, and the space‐occupying lesions. However, the two automatic and one semiautomatic liver volumetry methods developed may overcome this challenge. The 3DMIA, ASM, and PA methods all achieved accurate liver segmentation. They had good consistency of liver volumetry both in the normal and in the lesion group. These methods were comparable with other state‐of‐the‐art CT liver volumetry methods. Besides, the automatic ASM and PA liver volumetry methods were more efficient and have potential clinical use. The approach that employed priority information can greatly improve the accuracy and efficiency of segmentation, and facilitate the automatic method in clinical use.

## ACKNOWLEDGMENTS

This study was supported by grants from the following sources: the NSFC‐GD Union Foundation (No. U1401254), the National Key R&D Program (No. 2016YFC0106500), the Guangdong Science and Technology Project (Nos. 2015B020214005 and 2015A020214012), and the Shenzhen Key Science and Technology Development Project (Nos. JCYJ20140509174140681, JCYJ20140415162543027, JCYJ20150630114942306, and CXZZ20150430161339354).

## COPYRIGHT

This work is licensed under a Creative Commons Attribution 3.0 Unported License.

## APPENDICES

### Appendix A: The implementation procedure of 3DMIA

This semiautomatic interactive segmentation exploits a geodesic active contour model, according to image gray‐scale and spatial information, to delineate the liver boundary.^(18)^ The semiautomatic liver segmentation method may be summarized as follows:

1 (a) The image is preprocessed with a Gaussian smoothing filter, and the gradient image is computed.
2 (b) The initial contour for level set algorithm is extracted. The initial prediction is established by multiple iterations using region‐growing to make sure the curve is uniformly distributed inside the liver region. What's more, initial contour improves the efficiency of curve evolution of level set.
3 (c) The number of iteration of level set evolutions is set. Then the contour evolves automatically based on an evolution function. When the contour is inside the liver, the small gradient values make curve evolution expand rapidly towards the outside. Then, when the contour gets close to the boundary, the speed of evolution slows down. Finally, the evolution function reaches zero, and the outline stops evolving.
4 (d) When the contour arrives at the boundary or the algorithm reaches to maximum iterations, the evolving curve stops and the segmentation result is obtained. The semi‐automatic segmentation was then checked by slice by slice and the interactive segmentation toolkit (http://www.mitk.org) is used to refine the segmentation.

### Appendix B: The procedure of ASM‐based automatic volumetry

Before segmentation, a liver voxel classifier based on normalized liver intensity and location features was trained and later was used for fast liver presegmentation. A statistical shape model was constructed to capture global shape constraints and possible variations. An appearance model based on the Adaboost method was trained. In the online segmentation phase, liver pose and shape parameters are coarsely initialized by fitting the shape model to a distance map derived from the presegment result. Secondly, multiple iterations of the ASM model expand the model contour close to the liver boundary under the guidance of an appearance model. Finally, the final fitted model is free‐form deformed to the true liver boundary under the visual guidance of the appearance model. The coarse‐to‐fine strategy relaxes shape constraints and allows the model to deform appropriately to account for local imprecise offsets.

### Appendix C: The procedure of PA‐based automatic volumetry

PA is deformable registration of patient image to the reference atlas image with manual segmentation, constrained by integration of prior a probabilistic atlas image. The values of probabilistic atlas voxels are the given position's probabilities of belonging to the liver. To build a probabilistic atlas, all training image datasets are adjusted with same reference image using rigid transform to obtain uniform liver pose. With the guidance of the probabilistic atlas, the segmentation efficiently avoids undersegmentation (excluding hepatic lesions from liver area). However, fully automatic segmentation procedure requires liver localization. A statistical pose model (SPM) is deployed to detect livers on CT images. The SPM covers 98% of the variation of the observed liver poses (position and angle) and contributes to accurate prediction of the liver pose of the target image.

## Supporting information

Supplementary Material
