# Supplementary material for: Comparison of liver volumetry on contrast‐enhanced CT images: one semiautomatic and two automatic approaches
Source: J Appl Clin Med Phys. 2016 Nov 8;17(6):118–27. doi: 10.1120/jacmp.v17i6.6485 (PMC5690519; doi:10.1120/jacmp.v17i6.6485)
Supplement: Supplementary file 1 — Supplementary Material [file ACM2-17-118-s001.doc]

**Comparison of liver volumetry on contrast-enhanced CT images: one semi-automatic and two automatic approaches**

**Wei Cai**,^1,2a^ **Baochun He**,^1a^ **Yingfang Fan**,^2^ **Chihua Fang**,^2b^ **and** **Fucang Jia**^1b^

*Research Lab for Medical Imaging and Digital Surgery,^1^ Shenzhen Institutes of Advanced Technology, Chinese Academy of Sciences, Shenzhen 518055, China; Department of Hepatobiliary Surgery (I),^2^ Zhujiang Hospital, Southern Medical University, Guangzhou 510282, China*

*fangch_dr@126.com; fc.jia@siat.ac.cn*

Running title: Comparison of liver volumetry

^a^ These two authors contributed equally.

^b^ Corresponding author: Chihua Fang, Department of Hepatobiliary Surgery (I), Zhujiang University, Southern Medical University, Guangzhou 510282, China; phone: 86 (20) 6164 3208; fax: 86 (20) 62783398; email: fangch_dr@126.com; or Fucang Jia, Research Lab for Medical Imaging and Digital Surgery, Shenzhen Institutes of Advanced Technology, Chinese Academy of Sciences, Shenzhen, 518055, China; phone: 86 (755) 8639 2213; fax: 86 (755) 8639 2299; email: fc.jia@siat.ac.cn
